# Nitrite-mediated reduction of macrophage NADPH oxidase activity is dependent on xanthine oxidoreductase-derived nitric oxide but independent of S-nitrosation

**DOI:** 10.1016/j.redox.2016.09.015

**Published:** 2016-09-28

**Authors:** Christa Zollbrecht, A. Erik G. Persson, Jon O. Lundberg, Eddie Weitzberg, Mattias Carlström

**Affiliations:** a Department of Physiology and Pharmacology, Karolinska Institutet, Stockholm, Sweden; b Department of Medical Cell Biology, Uppsala University, Uppsala, Sweden

**Keywords:** DAF-FM DA, 4-amino-5-methylamino-2′,7′-difluorescein diacetate, DETA-NONOate, diethylenetriamine/nitric oxide adduct, Febuxostat, 2-(3-Cyano-4-isobutoxy-phenyl)-4-methyl-thiazole-5-carboxylic acid, CysNO, S-nitrosocysteine, L-NAME, Nω-nitro-l-arginine methylester hydrochloride, LPS, Lipopolysaccharides, NADH, β-Nicotinamide-adenine dinucleotide, NADPH, β-Nicotinamide-adenine dinucleotide 2′-phosphate, NO_2_^−^, nitrite, NO, nitric oxide, NOS, nitric oxide synthase, NOX, NADPH oxidase, ROS, reactive oxygen species, XOR, xanthine oxidoreductase, NADPH oxidase, Nitrite, Nitric oxide, Oxidative stress, S-nitrosation, Xanthine oxidoreductase

## Abstract

**Background:**

Inorganic nitrite has shown beneficial effects in cardiovascular and metabolic diseases partly via attenuation of NADPH-oxidase (NOX)-mediated oxidative stress. However, the exact mechanisms are still unclear. Here we investigated the role of S-nitrosation or altered expression of NOX subunits, and the role of xanthine oxidoreductase (XOR) in nitrite-derived nitric oxide (NO) production.

**Methods:**

Mouse macrophages were activated with LPS in the presence or absence of nitrite. NOX activity was measured by lucigenin-dependent chemiluminescence. Gene and protein expression of NOX2 subunits and XOR were investigated using qPCR and Western Blot. S-nitrosation of Nox2 and p22phox was studied with a Biotin Switch assay. Uric acid levels in cell culture medium were analyzed as a measure of XOR activity, and NO production was assessed by DAF-FM fluorescence.

**Results:**

NOX activity in activated macrophages was significantly reduced by nitrite. Reduced NOX activity was not attributed to decreased NOX gene expression. However, protein levels of p47phox and p67phox subunits were reduced by nitrite in activated macrophages. Protein expression of Nox2 and p22phox was not influenced by this treatment and neither was their S-nitrosation status. Increased uric acid levels after nitrite and diminished NO production during XOR-inhibition with febuxostat suggest that XOR is more active during nitrite-treatment of activated macrophages and plays an important role in the bioactivation of nitrite.

**Conclusions:**

Our findings contribute to the mechanistic understanding about the therapeutic effects associated with nitrite supplementation in many diseases. We show that nitrite-mediated inhibition of NOX activity cannot be explained by S-nitrosation of the NOX enzyme, but that changes in NOX2 expression and XOR function may contribute.

## Highlights

• Inorganic nitrite reduces NADPH oxidase activity in LPS-activated macrophages.
• Xanthine oxidoreductase plays an important role in nitrite reduction to NO.
• The effects of inorganic nitrite are not mediated by S-nitrosation of Nox2 and p22phox.
• Nitrite reduced protein expression of p47phox and p67phox.

## Graphical abstract

**Figure:**
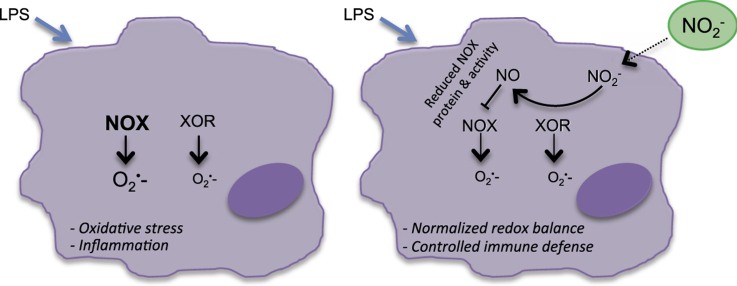

## 1 Introduction

Immune cells, like macrophages, are major sources of reactive oxygen species (ROS) in the body. Particularly superoxide is produced by NADPH oxidase (NOX), an enzyme family consisting of several isoforms of which NOX2 is the main isoform expressed in macrophages and neutrophils. The superoxide anion is important for effective host defense, immune cell function and is a component of the cellular redox signaling. However, when overproduction of superoxide persists, the normal redox signaling gets disrupted leading to oxidative stress, in which an imbalance develops between ROS and nitric oxide (NO). Oxidative stress represents a major risk factor and cause of both metabolic and cardiovascular diseases [1]. It can be ameliorated either by a decrease in ROS production or an increase in NO bioavailability. In addition to NO production via the classic nitric oxide synthase (NOS) pathway, fueling the alternative nitrate-nitrite-NO pathway with inorganic nitrate and nitrite has shown beneficial effects in many disorders and experimental disease models via restoring NO bioavailability [2], [3], [4]. However, it is still not clear how this effect is mediated and signaling events may occur in a cell-, tissue- and environment-specific manner. In a recent study, we demonstrated that augmented NOX-mediated ROS production in lipopolysaccharide (LPS)-activated mouse macrophages was almost normalized by simultaneous nitrite treatment [5]. This effect of nitrite on superoxide production was abolished by an NO scavenger and also during inhibition of xanthine oxidoreductase (XOR), which suggests that nitrite acts via an NO signaling mechanism that is dependent on functional XOR.

XOR, a molybdoflavin-containing enzyme responsible for the terminal steps of purine catabolism, catalyzes the hydroxylation of hypoxanthine to xanthine and xanthine to uric acid, which is accompanied by ROS production. However, under certain conditions, XOR is able to use inorganic nitrite as a substrate and generate NO [6], [7]. Especially during low oxygen tensions, which occur during ischemia reperfusion injuries, this way of NO production seems important and may compensate for a compromised NOS function since this enzyme is dependent on oxygen. Immune cells like macrophages are present at sites of tissue injury where they may become lytic and therefore a significant source of XOR.

In addition to activating cGMP-dependent signaling pathways NO can mediate protein S-nitrosation by attachment of an NO^+^ moiety to cysteine residues in proteins. This S-nitrosothiol formation may influence protein function and has been widely recognized as a signaling mechanism [8], [9]. There is emerging evidence that protein S-nitrosation may be altered during disease development, and hence play a role in renal and cardiac protection [10] as well as in modulation of the immune system [11]. The reaction is highly regulated and exhibits remarkable specificity occurring only on selected cysteine residues located within a certain amino acid motif and basic surrounding conditions [12].

In the current study we used murine macrophages to investigate if the previously demonstrated effects of inorganic nitrite on oxidative stress are achieved via XOR-dependent NO production and if NO-mediated S-nitrosation of NOX subunits may contribute to the reduced NOX activity following treatment with inorganic nitrite.

## 2 Material and methods

### 2.1 Materials

Unless otherwise indicated all chemicals were purchased from Sigma-Aldrich (Stockholm, Sweden).

### 2.2 Cell culture

Mouse peritoneal macrophages (IC-21 ATCC TIB-186; American Type Culture Collection, Manassas, VA, USA) were cultured in RPMI 1640 medium complemented with 10% fetal bovine serum, 2 mmol/l l-glutamine, 100 units/ml penicillin, and 100 µg/ml streptomycin (all Thermo Fisher Scientific, Stockholm, Sweden) and in humidified air with 5% CO_2_ at 37 °C. Medium was replaced every other day and cells were passaged using Dulbecco's phosphate-buffered saline (DPBS, without Ca^2+^/Mg^2+^) when reaching 80–90% confluency. For experiments, macrophages were seeded in plates at a density of 500000 cells/ml and allowed to attach overnight.

### 2.3 Pharmacological treatments

Pharmacological treatments of macrophages were performed in RPMI medium without fetal bovine serum. After the normal growth medium was removed, cells were washed once with DPBS. Macrophages were then incubated with sodium nitrite (NaNO_2_, 10 µmol/l), LPS from *Escherichia coli* endotoxin (0111:B4, 10 ng/ml), diethylenetriamine/NO adduct (DETA-NONOate, 0.5 mmol/l), N_ω_-nitro-l-arginine methylester hydrochloride (l-NAME, 1 mmol/l), febuxostat (30 nmol/l) or a combination thereof, for 24 h or the indicated times. l-NAME and febuxostat were added 30 min before treatment with other substances.

To prepare S-nitrosocysteine (CysNO), 1 vol of 200 mmol/l l-cysteine (in 1 mol/l HCl) was incubated with 1 vol of 200 mol/l sodium nitrite (in distilled water) for 30 min at room temperature in the dark. The solution was neutralized by the addition of 2 volumes of 1 mol/l K_2_HPO_4_ (pH 7.4) buffer and was stored in aliquots at −80 °C until use. The concentration of formed CysNO was determined from the optical absorbance at 338 nm by using the extinction coefficient 900/(mol*l*cm) [13]. After LPS-treatment or incubation in serum-free medium for 24 h, macrophages were washed twice with phosphate buffered saline (PBS, containing Ca^2+^/Mg^2+^) followed by incubation with CysNO in PBS for 15 min at 37 °C. Subsequently, cells were washed with PBS twice and collected for Biotin Switch assay (see below).

### 2.4 NADPH-dependent superoxide production

Lucigenin-dependent chemiluminescence was used to determine NADPH oxidase activity, measured as NADPH-dependent superoxide production, as described previously [5]. After pharmacological treatments, macrophages were washed with DPBS and detached from the culture dish by incubation in DPBS at 37 °C for 15 min. Cell suspensions were transferred to measurement tubes and incubated with dark-adapted lucigenin (5 µmol/l) at 37 °C for 15 min. Superoxide production was started by the injection of the NOX substrate NADPH (100 µmol/l) and the chemiluminescence signal was measured every 3 s for 3 min with an AutoLumat LB953 Multi-Tube Luminometer (Berthold Technologies, Bad Wildbad, Germany). Previous studies have demonstrated that incubation with either tempol to scavenge superoxide or VAS3947 to inhibit NOX reduces the chemiluminescence signal with approximately 90% in both cell culture and tissues [14], [15], suggesting that mainly superoxide from NOX is measured. Moreover, unspecific lucigenin-mediated superoxide generation in the absence of added NADPH was similar to the blank (i.e. PBS only), and should therefore not have any significant impact on the results.

### 2.5 Biotin Switch assay

Protein S-nitrosation levels were determined in macrophage lysates using the Biotin Switch assay with some modifications from the original protocol [16]. All steps before the neutravidin-purification of biotinylated proteins were carried out in the dark. After treatments, macrophages were collected and lysed by scraping in TENT buffer (50 mmol/l Tris-HCl pH 7.2, 150 mmol/l NaCl, 1 mmol/l EDTA, 0.1 mmol/l neocuproine, 1% Triton X-100, protease inhibitor cocktail) and incubation on ice for 15 min. The soluble fraction was obtained by centrifugation at 10000 g and 4 °C for 15 min. Protein concentration was determined using the Bradford protein assay (Bio-Rad) and samples were adjusted to 0.5 mg/ml with TEN buffer (TENT without Triton X-100). Free thiols were blocked in 4 volumes of HENS buffer (250 mmol/l Hepes pH 7.7, 1 mmol/l EDTA, 0.1 mmol/l neocuproine, 1% SDS) containing 20 mmol/l *S*-methyl methanethiosulfonate at 50 °C for 30 min with agitation every 5 min. Proteins were precipitated with cold acetone for at least 30 min at −20 °C to remove excess blocking reagent. To reduce SNO-thiols and label resulting SH-residues, precipitated proteins were resuspended in 300 µl HENS/mg protein containing 5 mmol/l ascorbate and incubated in the presence of 1 mmol/l (N-[6-(Biotinamido)hexyl]-3′-(2′-pyridyldithio)propionamide (HPDP-biotin, Thermo Fisher Scientific) for 2 h at room temperature. After aceton precipitation, biotinylated proteins were resuspended in 150 µl HENS/mg protein and purified by incubation with NeutrAvidin Plus Ultra Link Resin (Thermo Fisher Scientific) in 5 volumes neutralization buffer (20 mmol/l Hepes pH 7.7, 100 mmol/l NaCl, 1 mmol/l EDTA, 0.5% Triton X-100) over night at 4 °C. Bound proteins were washed extensively (20 mmol/l Hepes pH 7.7, 600 mmol NaCl, 1 mmol/l EDTA, 0.5% Triton X-100), eluted from the resin at 37 °C for 20 min with 1 mmol/l β-mercaptoethanol in 20 mmol/l Hepes pH 7.7, 100 mmol/l NaCl, 1 mmol/l EDTA, and analyzed using Western Blot.

### 2.6 Real-time PCR

The mRNA expression levels of NOX subunits and xanthine oxidase (XOR) were determined by real-time PCR. After treatments, macrophages were collected in β-mercaptoethanol containing RLT buffer and total RNA was isolated using the RNeasy Mini Kit (Qiagen, Sollentuna, Sweden) according to the manufacturer's instructions. RNA purity and concentration were assessed spectrophotometrically on the Nanodrop ND-1000 (Thermo Fisher Scientific). Equal amounts of RNA were reverse transcribed to cDNA with the High Capacity cDNA Reverse Transcription Kit (Thermo Fisher Scientific). Real-time PCR was performed in 96-well plates in a total reaction volume of 20 µl on an ABI 7500 Real-Time PCR System using Power SYBR Green Master mix (Thermo Fisher Scientific) and gene specific primers (Table 1). Mean x-fold relative gene expression changes were calculated with the ∆∆Ct-method [17], standardizing the results to the housekeeping gene β-actin.

### 2.7 Western Blot

Protein fractions from the Biotin Switch assay (input and eluted proteins) were denaturated in loading buffer (62.5 mmol/l Tris-HCl pH 6.8, 10% glycerol, 2% sodium dodecyl sulfate (SDS), 0.01% bromophenol blue, 0.8% β-mercaptoethanol) at 95 °C for 5 min. Protein expression of NOX subunits was measured by Western Blot in macrophages. Whole cell lysates were prepared by scraping the cells in lysis buffer (10 mmol/l Tris-HCl pH 8, 150 mmol/l NaCl, 5 mmol/l EDTA, 60 mmol/l N-octyl-glucoside, 1% Triton X-100, protease inhibitor cocktail) followed by centrifugation at 10000 g for 15 min. Protein concentration was determined in the soluble fraction using the Bradford protein assay (Bio-Rad, Solna, Sweden) and equal amounts of protein were denaturated in loading buffer at 95 °C for 5 min. Proteins were separated on 4–20% SDS-polyacrylamide gels (Bio-Rad) using electrophoresis and transferred to polyvinylidene difluoride membranes (Bio-Rad). The membranes were blocked in 5% nonfat dry milk containing TBS-T (20 mmol/l Trizma base pH 7.6, 150 mmol/l NaCl, 0.1% Tween-20), followed by incubation with primary antibodies (gp91phox/Nox2, BD Biosciences, Stockholm, Sweden; p67phox, Cell Signaling/BioNordika, Stockholm, Sweden; p22phox, p47phox and XOR, Santa Cruz, Heidelberg, Germany). Primary antibodies were detected using the respective horseradish peroxidase-conjugated goat anti-rabbit or anti-mouse IgG (Cell Signaling). Protein bands were visualized by Clarity Western ECL Substrate (Bio-Rad) and intensities were quantified using densitometry (Image Lab software, Bio-Rad). To ensure equal protein loading, membranes were stripped using Restore™ PLUS Western Blot Stripping Buffer (Thermo Fisher Scientific) and after blocking re-probed with primary antibody against β-actin (Santa Cruz) and anti-mouse IgG. Mean x-fold relative protein expression changes were calculated by standardizing the results to the housekeeping protein β-actin. For the comparison of S-nitrosated proteins between LPS- and LPS+CysNO treated cells, the relative intensity ratio SNO-protein/input was calculated for Nox2 and p22phox, respectively, and is presented in the text (LPS treated groups=1). Since the LPS-treatment strongly affected the expression of the studied proteins and SNO-signals were often weak, quantification of SNO-protein/input seemed arbitrary and was omitted for other between-group comparisons but representative blots are shown instead.

### 2.8 Uric acid assay

Uric acid is the final oxidation end product of purine nucleotide metabolism catalyzed by XOR from xanthine and hypoxanthine. Uric acid levels were measured in macrophage cell culture medium after 24 h treatments as a measure of XOR activity. Samples were incubated with 80 µmol/l Amplex Ultra Red reagent (Thermo Fisher Scientific), 0.8 U/ml horse radish peroxidase and 2 U/ml uricase in 100 mmol/l Tris-HCl pH 7.5 for 30 min at 37 °C. The fluorescence signal was measured in a microplate reader (excitation 530 nm, emission 590 nm) and the concentration of uric acid was calculated from a standard curve on the same plate. To investigate how much of the uric acid is derived from XOR, uric acid levels in culture medium of febuxostat (30 nmol/l) treated macrophages were measured with the same approach. As a control, 2.5 mU/ml purified XOR enzyme was used in a cell-free approach and incubated for 30 min at 37 °C with 32 µmol/l hypoxanthine (Sigma), 80 µmol/l Amplex Ultra Red reagent (Thermo Fisher Scientific), 0.8 U/ml horse radish peroxidase in 100 mmol/l Tris-HCl pH 7.5 with or without 2 U/ml uricase and in the presence of different febuxostat concentrations (3, 30, 300, 3000 nmol/l). The fluorescence signal was measured in a microplate reader (excitation 530 nm, emission 590 nm) and the signal without uricase (representing H_2_O_2_ production) was subtracted from the signal with uricase (resulting in only uric acid-derived fluorescence signal). Results are presented as % inhibition with febuxostat vs. no febuxostat.

### 2.9 DAF-FM fluorescence

The cell-permeable fluorescent NO indicator 4,5-diaminofluorescein-FM diacetate (DAF-FM DA) was used to detect NO production in macrophages. The cells were plated and treated in 96-well plates as described above. After 24 h, the cells were loaded with DAF-FM DA (10 µM) for 45 min at 37 °C. Cells were then washed with DPBS and transferred to a black 96-well plate. The fluorescence signal was measured in a microplate reader (excitation 495 nm, emission 515 nm) and is expressed as the percentage change of the l-NAME treated cells.

### 2.10 Statistical analysis

Results are expressed as means±SEM. Statistical analyses were performed using one-way ANOVA followed by Bonferroni's multiple comparison test (GraphPad Prism version 5.04, San Diego, CA, USA). A p-value<0.05 was considered statistically significant.

## 3 Results

### 3.1 Nitrite reduces superoxide generation in LPS-activated macrophage

**Fig. 1:**
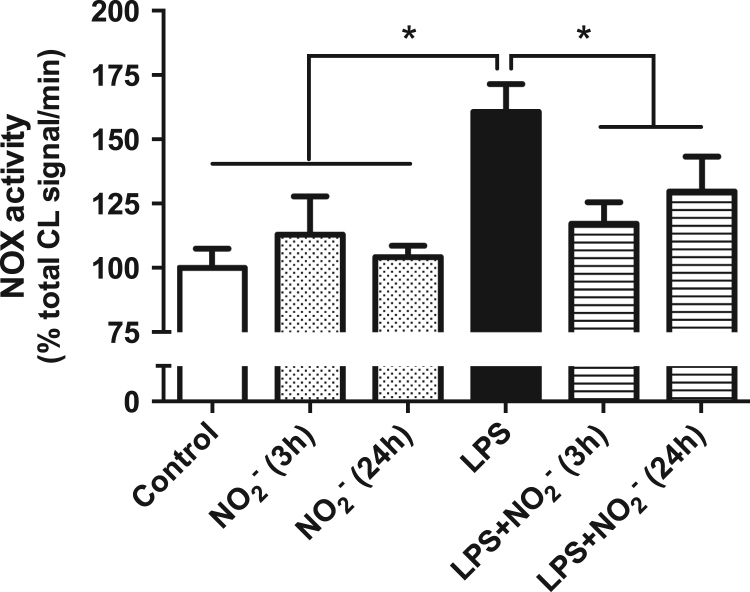
NADPH-dependent superoxide production. NADPH-oxidase-derived superoxide production is presented as % chemiluminescence (CL) signal/min of the nonactivated control macrophages. Data are shown as mean±SEM, n=4–8/group, * p<0.05 between indicated groups.

As has been shown recently [5], 24 h activation of macrophages with LPS resulted in increased NADPH-dependent superoxide production and this was attenuated by simultaneous incubation with nitrite. To mimic a more therapeutic situation with administration of nitrite during ongoing inflammation, macrophages were incubated with LPS for 21 h, then nitrite was added and the cells were incubated for another 3 h. This short time was sufficient for a significant reduction of the superoxide generation even though LPS was still present (Fig. 1). In control cells, nitrite had no effect on superoxide production independently of the incubation time.

### 3.2 Nitrite does not alter the gene expression of NOX2 subunits

**Fig. 2:**
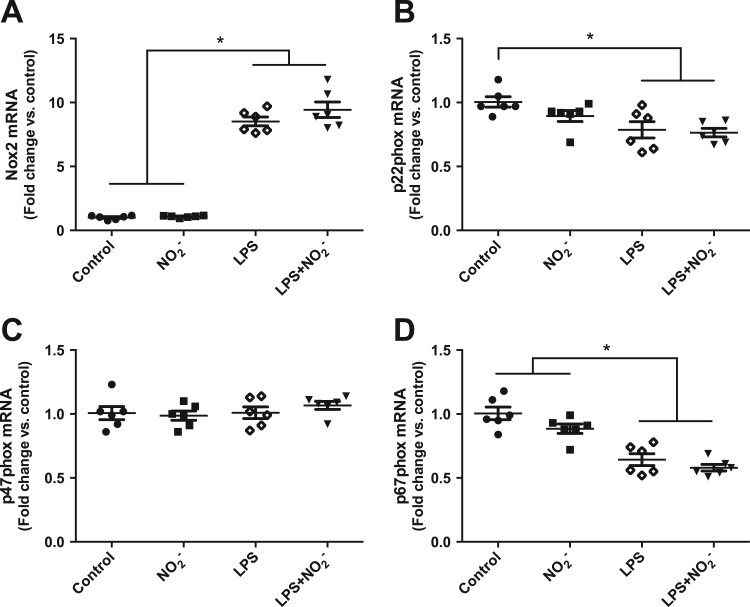
Gene expression of NOX subunits. Relative mRNA levels for the NOX2 subunits Nox2, p22phox, p47phox and p67phox are presented as fold change of the nonactivated control macrophages for each gene. Data are shown as mean±SEM, n=6/group, * p<0.05 between indicated groups.

One possible mechanism how nitrite might mediate this attenuation of NOX-derived superoxide formation is through changes in enzyme expression. Therefore, we investigated gene expression levels of the NOX2 subunits in activated and nonactivated macrophages. Treatment with LPS resulted in a strong induction of Nox2, however p47phox was unchanged and levels of p22phox and p67phox were even decreased. Nitrite had no effect in nonactivated cells and did not alter the expression levels of any of the analyzed subunits when administered together with LPS compared to LPS alone (Fig. 2A–D).

### 3.3 Nitrite modulates protein expression of NOX2 subunits in LPS-activated macrophages

**Fig. 3:**
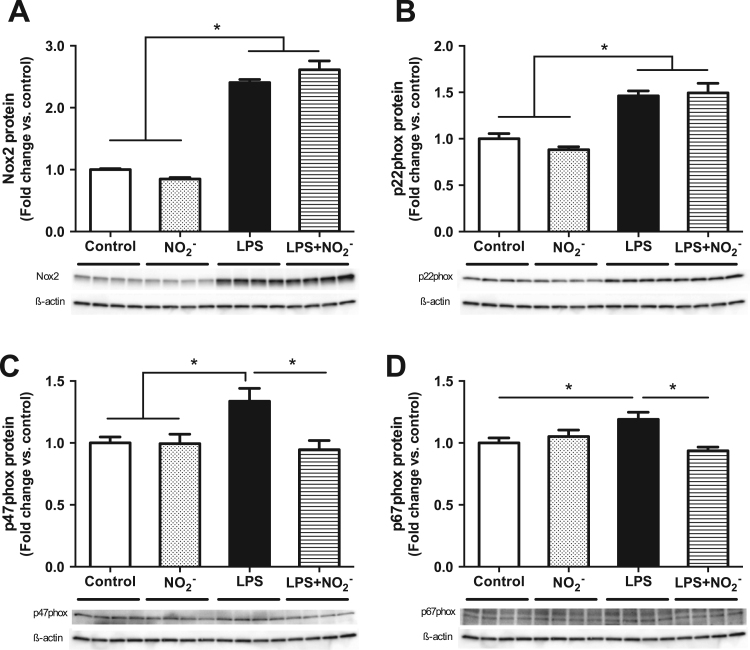
Protein expression of NOX subunits. Relative protein levels for the NOX2 subunits Nox2, p22phox, p47phox and p67phox are presented as fold change of the nonactivated control macrophages for each protein. Data are shown as mean±SEM, n=8/group, * p<0.05 between indicated groups.

Since mRNA levels do not always reflect the abundance of protein in the cells, we also analyzed the protein expression of NOX2 subunits in activated and nonactivated macrophages. Stimulation of the cells with LPS led to a significant increase of Nox2, p22phox, p47phox and p67phox. Whereas the levels of the membrane bound subunits Nox2 and p22phox were not influenced by the presence of nitrite (Fig. 3A,B), the expression of the cytosolic subunits p47phox and p67phox was significantly reduced in cells treated with LPS plus nitrite compared with LPS alone (Fig. 3C,D).

### 3.4 Nitrite does not mediate S-nitrosation of NOX2 subunits

**Fig. 4:**
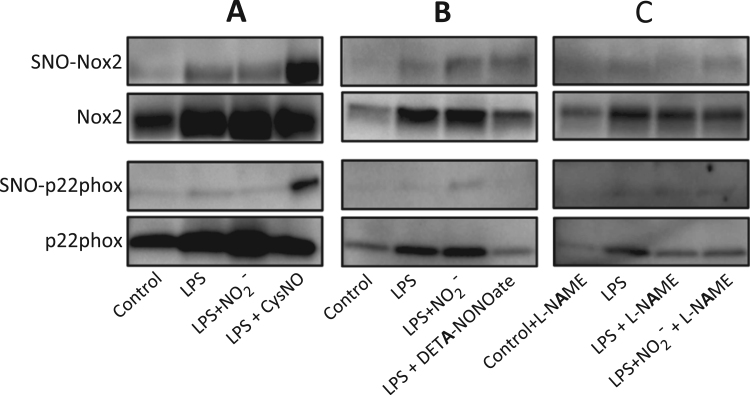
S-nitrosation of NOX subunits. S-nitrosated proteins (SNO) and the input of Nox2 and p22phox are shown. S-nitrosation was analyzed after cell treatment with LPS, LPS plus nitrite and either a quick-releasing NO donor (A), a slow-releasing NO-donor (B) or NOS-inhibition (C). Representative results from independent experiments are shown.

S-nitrosation of cysteine residues has been described as a posttranslational modification which impacts on the activity of many proteins. We therefore investigated if nitrite-derived NO mediates S-nitrosation of the membrane bound NOX2 subunits and thereby contributes to the reduced activity of NOX in LPS-activated macrophages. In cells treated with LPS, a proportion of both Nox2 and p22phox underwent S-nitrosation (relative intensity ratio SNO-protein/input 1 for NOX2 and 1 for p22phox; see methods for details); however, simultaneous incubation with nitrite for 24 h did not increase this proportion (relative intensity ratio SNO-protein/input 0.9 for Nox2 and 0.9 for p22phox). In contrast, a short incubation (15 min) with the NO-donor CysNO resulted in clear S-nitrosation of both Nox2 and p22phox (relative intensity ratio SNO-protein/input 3.3 for Nox2 and 6.5 for p22phox), which demonstrates that these proteins are able to undergo S-nitrosation under certain conditions (Fig. 4A). The mechanisms and kinetics of the release of NO and other reactive nitrogen species vary between different types of NO-donors depending on their decomposition and the requirement of specific conditions or catalysts [18]. Therefore, macrophages were incubated with the slow-releasing NO-donor DETA-NONOate for 24 h in the presence of LPS, which resulted in a similar level of S-nitrosation of Nox2 compared to nitrite, whereas p22hox was not S-nitrosated (Fig. 4B). Especially the inducible form of endogenous NO synthases (iNOS) produces high amounts of NO in response to LPS-activation of macrophages. To exclude the possibility that this large amount of iNOS-derived NO would mask an effect of nitrite in the detection of S-nitrosation levels, macrophages were treated with l-NAME to block all NOS isoforms. However, even during NOS inhibition there were no differences between cells treated with LPS alone and LPS plus nitrite (Fig. 4C). Taken together, these results indicate that the ability of nitrite to reduce superoxide production during LPS-activation of macrophages is not mediated by S-nitrosation of Nox2 or p22phox.

### 3.5 The effects of nitrite are dependent on XOR

**Fig. 5:**
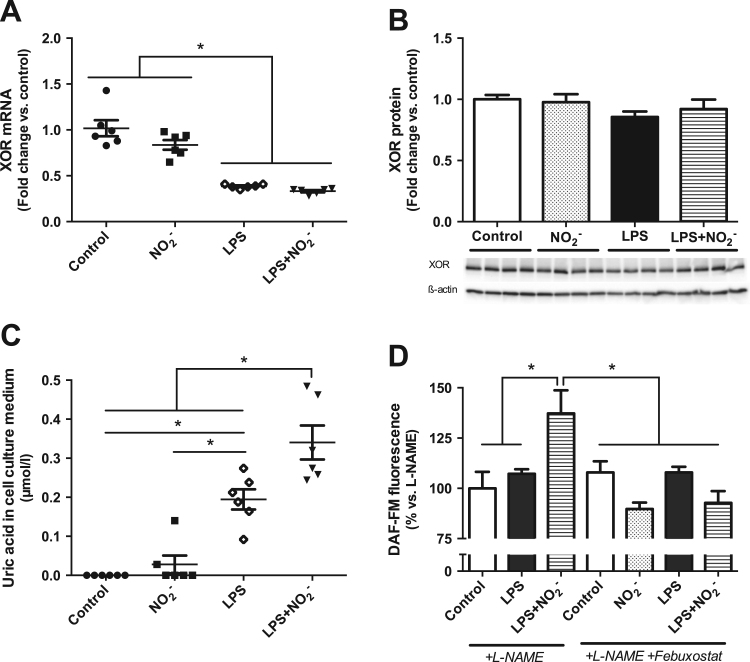
Role of XOR in the effects mediated by nitrite. Relative mRNA (A) and protein levels (B) of XOR are presented as fold change of nonactivated control macrophages. Uric acid concentration in macrophage culture medium as a measure of XOR activity is given in µmol/l (C). NO production was measured as DAF-FM fluorescence and is presented as % of the l-NAME treated macrophages. Data are shown as mean±SEM, n=6/group (A,C), n=4/group (B), n=8/group (D), * p<0.05 between indicated groups.

We have previously demonstrated that inhibition of XOR abolished the effect of nitrite on NOX-mediated superoxide production in LPS-activated macrophages [5]. Since there is evidence implicating XOR in the reduction of nitrite to NO and other bioactive nitrogen species [7], [19], we investigated the role of this enzyme further by measuring its gene and protein expression as well as its activity in macrophages. While mRNA levels of XOR were reduced both upon LPS activation and LPS plus nitrite treatment, the protein expression was not influenced by any of the treatments (Fig. 5A,B). The levels of uric acid were analyzed in the cell culture medium after 24 h treatment of macrophages as a measure of XOR activity. As a control, uric acid production was measured in the presence of febuxostat and resulted in 50–70% inhibition both in culture medium of febuxostat treated macrophages and using purified XOR enzyme (Supplemental Figure 1). LPS-activation resulted in an increase in uric acid production compared to nonactivated cells. Simultaneous treatment with nitrite increased uric acid levels even further in activated macrophages, which indicates an induction of XOR activity under these conditions (Fig. 5C). To examine if this change in XOR activity is relevant for nitrite-derived NO production, we measured DAF-FM fluorescence, after 24 h treatment of the macrophages. As shown previously [5], the increased NO production upon treatment with LPS plus nitrite could not be blocked by NOS-inhibition with l-NAME. However, additional simultaneous inhibition of XOR with febuxostat significantly reduced the NO production, resulting in control levels and was similar in all other groups (Fig. 5D). These results strongly suggest that XOR, not through changes in its expression but its activity, is involved in the reduction of nitrite to NO and the subsequent inhibition of NOX-derived superoxide production in activated macrophages.

## 4 Discussion

We demonstrate here that nitrite-mediated attenuation of NOX-derived superoxide production in LPS-activated macrophages is associated with XOR-dependent NO production. Furthermore, we found that nitrite-mediated reduction of superoxide production does not involve S-nitrosation of the membrane-bound NOX subunits Nox2 and p22phox, but can partially be attributed to changes in protein expression of the cytosolic subunits p47phox and p67phox.

According to current knowledge, phagocytic NADPH oxidase NOX2, which is the main isoform expressed in macrophages, consists of two membrane bound subunits Nox2 (or gp91phox) and p22phox, the cytosolic subunits p47phox, p67phox, p40phox and a small GTPase Rac. The assembly process of the active enzyme complex has been described in detail [20]. Briefly, upon stimulation, p47phox is phosphorylated and translocates, in complex with p67phox and p40phox, to the cell membrane where the active enzyme complex is formed. This results in a burst of superoxide production. Looking at the gene expression of NOX subunits we found a strong induction only of Nox2 mRNA in LPS-activated macrophages. In contrast, the protein levels of all four investigated subunits were significantly increased after LPS stimulation. Interestingly, the simultaneous treatment with nitrite only showed an effect on protein expression and reduced levels of p47phox and p67phox, but not Nox2 and p22phox. If this extent of protein reduction has sufficient impact on the enzyme activity to explain the reduced superoxide production seen with nitrite in LPS-activated macrophages is unclear. In addition to expression levels, also the intracellular distribution of these subunits plays an important role for NOX activity and might be altered by nitrite. However, it has been shown in human neutrophils that only 10–15% of p47phox (in complex with p67phox) translocates to the membrane upon cell activation [21], [22]. Future studies are warranted to investigate if nitrite may influence these translocation processes, and hence contribute to the reduced NOX activity.

Supplementation with both inorganic nitrate and nitrite have shown beneficial effects in various diseases and experimental models, e.g. reduced blood pressure in healthy volunteers [23] and in hypertensive patients [24], [25] and rats [26], reversed features of metabolic syndrome [27] and improved glucose and insulin homeostasis in type 2 diabetes and its complications [28], [29]. In many cases, the protective effects of nitrate and nitrite could be linked to reduced NOX-derived oxidative stress [29], [30], [31]. In the current study, we show a therapeutic effect with nitrite, i.e. administration after 21 h LPS challenge resulted in reduced NOX activity and prevention of oxidative stress. Also in vivo nitrite has shown protective effects not only against LPS- but also against tumor necrosis factor-induced shock in mice [32], however, the effects on NOX were not addressed in that study.

Since nitrite abolished superoxide production by NOX in an NO-dependent manner [5], signaling via NO became of particular interest. There are three principal pathways of physiological NO signaling [33] including the reaction of NO with thiyl radicals to form S-nitrosothiols. S-nitrosation has gained increased recognition as a functionally important posttranslational modification and might provide protection by preventing irreversible oxidative thiol-modifications by ROS or peroxynitrite [34]. This action suggests a potential for nitrite-derived NO to S-nitrosate NOX subunits. Most studies of proteins that have been identified as substrates for S-nitrosation used greatly simplified in vitro preparations and/or relied on exogenous NO donors rather than physiological NO produced by endogenous enzymes. We support here the discrepancy between such conditions since we found that in our experimental conditions nitrite-derived NO did not mediate S-nitrosation of Nox2 and p22phox to a greater extent than incubation of macrophages with LPS alone. However, the presence of the NO donor CysNO resulted in strong S-nitrosation of both proteins. These results indicate that nitrite does not reduce LPS-induced superoxide production in these cells via functional NO-mediated modulation of the membrane-bound NOX subunits. However, non-physiologically high NO concentrations resulted in S-nitrosation of both Nox2 and p22phox. There are studies suggesting S-nitrosation of NOX isoforms and subunits, but in these studies purified proteins and/or NO donors were used [35], [36] and the physiological relevance remains to be proven. The activity of Nox5, an isoform only expressed in humans, has been shown to be reduced via NO-mediated S-nitrosation in transfected COS-7 cells and four cysteine residues have been identified as targets for S-nitrosation [37]. This could be of particular interest for the favorable effects observed with nitrite in humans and future studies investigating the signaling mechanism in humans are warranted.

XOR has been described as damaging during disease conditions like ischemia reperfusion injury or atherosclerotic plaques due to its enhanced affinity for oxygen and concomitant ROS production when performing its generally accepted role in purine catabolism [38], [39]. In addition, the detailed mechanism has been elucidated how XOR can also catalyze the reduction of nitrite to NO using either NADH or xanthine as reducing substrate [40], [41]. Especially during conditions with low oxygen tension, XOR-mediated NO production is fairly high and can compensate for impaired NOS-derived, oxygen-dependent NO synthesis [4]. Also in normoxia XOR has been found to produce NO when nitrite is present as substrate [5], [7], [42]. Nitrite-mediated attenuation of superoxide production in LPS-activated macrophages has been described as XOR-dependent since the effect was abolished in the presence of the XOR inhibitor febuxostat [5]. In this study, we demonstrate that also the NO production in LPS-activated macrophages was dependent on XOR and could be blocked by febuxostat whereas this effect was independent of NOS. We found increased uric acid levels in the cell culture medium, which reflect increased XOR activity and are in agreement with previous findings showing increased XOR activity in human macrophages upon LPS-stimulation [43]. In contrast to other tissue and cell types [44], [45] gene expression of XOR was not increased upon inflammatory stimulation. In the current study we show that protein levels of XOR were not influenced by any of the treatments and the gene expression was even decreased during treatment with LPS and LPS plus nitrite, compared to the observed induction of NOX expression. Therefore we conclude that the elevated XOR activity does not contribute to the observed superoxide levels, but rather that NOX is the main source. On the contrary, the increased uric acid formation might reflect increased nitrite reductase activity of XOR, which leads to increased NO bioavailability and subsequently reduced NOX-derived superoxide. Still, it is unclear how nitrite-derived NO and the reduced NOX activity are connected. Direct scavenging of ROS by NO [5] as well as enzyme modification via S-nitrosation, as shown here, seem unlikely.

In conclusion, we show in LPS-activated macrophages that XOR plays an important role in nitrite reduction to NO, leading to decreased NOX-derived superoxide generation. Reduced NOX activity is however not mediated by any significant NO-dependent S-nitrosation of the membrane-bound NOX subunits Nox2 or p22phox. Instead, our findings suggest that a reduced protein expression of the cytosolic NOX subunits may contribute to the favorable effect of nitrite. These mechanistic details are important to further characterize inorganic nitrate and nitrite as potential therapeutics in ROS-mediated cardiovascular and metabolic diseases.

## Conflict of interests

Lundberg and Weitzberg are co-inventors on patent applications related to the therapeutic use of inorganic nitrate.

## Figures and Tables

**Table 1:** Nucleotide-sequences of primers used in real-time PCR.

Gene	Forward primer (5′−3′)	Reverse primer (5′−3′)
*Nox2*	GCACCTGCAGCCTGCCTGAATT	TTGTGTGGATGGCGGTGTGCA
*p22phox*	CTGGCGTCTGGCCTGATTCTCATC	CCGAAAAGCTTCACCACAGAGGTCA
*p47phox*	CAGCCATGGGGGACACCTTCATT	GCCTCAATGGGGAACATCTCCTTCA
*p67phox*	AAGACCTTAAAGAGGCCTTGACGCA	TCGGACTTCATGTTGGTTGCCAA
*XOR*	CAGCATCCCCATTGAGTTCA	GCATAGATGGCCCTCTTGTTG
*β*-*actin*	GCTCCTCCTGAGCGCAAT	GTGGACAGTGAGGCCAGGAT
