# Supplementary material for: Nitrite-mediated reduction of macrophage NADPH oxidase activity is dependent on xanthine oxidoreductase-derived nitric oxide but independent of S-nitrosation
Source: Redox Biol. 2016 Sep 28;10:119–27. doi: 10.1016/j.redox.2016.09.015 (PMC5065649; doi:10.1016/j.redox.2016.09.015)
Supplement: Supplementary file 1 — Supplementary material [file mmc1.pdf]

Nitrite-mediated reduction of macrophage NADPH oxidase activity is dependent on xanthine oxidoreductase-derived nitric oxide but independent of S-nitrosation

Christa Zollbrecht[a,b], A. Erik G. Persson[b], Jon O. Lundberg[a], Eddie Weitzberg[a], Mattias Carlström[a*]

[a] Department of Physiology and Pharmacology, Karolinska Institutet, Stockholm, Sweden

[b] Department of Medical Cell Biology, Uppsala University, Uppsala, Sweden

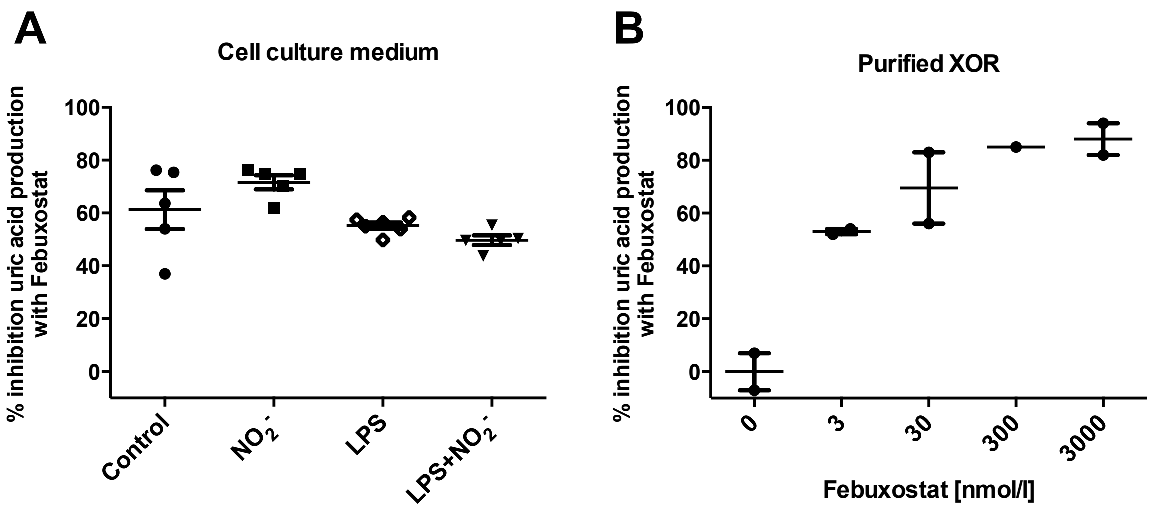

Supplemental Figure 1: Uric acid produced by XOR is blocked by febuxostat. Culture medium of febuxostat treated macrophages showed 50-70 % inhibition of uric acid production (vs. no febuxostat) (A). Using purified XOR enzyme, febuxostat reduced uric acid production dose-dependently and % inhibition was similar to that observed in cells when using the same concentration of 30 nmol/l (B).
